# Hallmarks of cancer—the new testament

**DOI:** 10.1098/rsob.200358

**Published:** 2021-01-20

**Authors:** Sasi S. Senga, Richard P. Grose

**Affiliations:** Centre for Tumour Biology, Barts Cancer Institute, Queen Mary University of London, London EC1M 6BQ, UK

**Keywords:** cancer hallmarks, epigenetics, de-differentiation, microbiome, neuronal signalling

## Abstract

Diagnosis and treatment of disease demand a sound understanding of the underlying mechanisms, determining any Achilles' heel that can be targeted in effective therapies. Throughout history, this endeavour to decipher the origin and mechanism of transformation of a normal cell into cancer has led to various theories—from cancer as a curse to an understanding at the level of single-cell heterogeneity, meaning even among a single sub-type of cancer there are myriad molecular challenges to overcome. With increasing insight into cancer genetics and biology, the disease has become ever more complex to understand. The complexity of cancer as a disease was distilled into key traits by Hanahan and Weinberg in their seminal ‘Hallmarks of Cancer' reviews. This lucid conceptualization of complex cancer biology is widely accepted and has helped advance cancer therapeutics by targeting the various hallmarks but, with the advancement in technologies, there is greater granularity in how we view cancer as a disease, and the additional understanding over the past decade requires us to revisit the hallmarks of cancer. Based on extensive study of the cancer research literature, we propose four novel hallmarks of cancer, namely, the ability of cells to regress from a specific specialized functional state, epigenetic changes that can affect gene expression, the role of microorganisms and neuronal signalling, to be included in the hallmark conceptualization along with evidence of various means to exploit them therapeutically.

## Graphical abstract

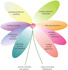

## 1. A historical perspective on cancer

Ancient Egyptians believed cancer to be a curse, as seen from evidence as old as 3000 BC from the Edwin Smith Papyrus which described breast cancer [1] and Ebers Papyrus dated 1500 BC which described skin, uterus and other types of tumours [2]. Hippocrates proposed the idea of an excess of black bile to be the reason for cancer, the idea was further developed by Greek physician Galen, revered physician to the Emperor Marcus Aurelius, who suggested black bile caused incurable types of cancer while yellow bile caused curable variants of cancer [3]. This was refuted in the sixteenth century by the renowned anatomist Andreas Vesalius, who disproved the existence of black bile [4]. In the sixteenth century, Paracelsus identified the first correlation between cancer and the environment, showing deposits of arsenic salts and sulfur in the blood of mine workers were associated with cancer. This laid the foundation for later work by others, namely Percival Pott (chimney sweeps), John Hill (snuff) and Ludwig Rehn (aniline dyes) ([5]. In 1914, Theodor Boveri was the first to hypothesize that abnormal segregation of chromosomes to daughter cells can lead to tumour development in ‘*Zur Frage der Entstehung Maligner Tumoren*' [6].

**Figure 1.**
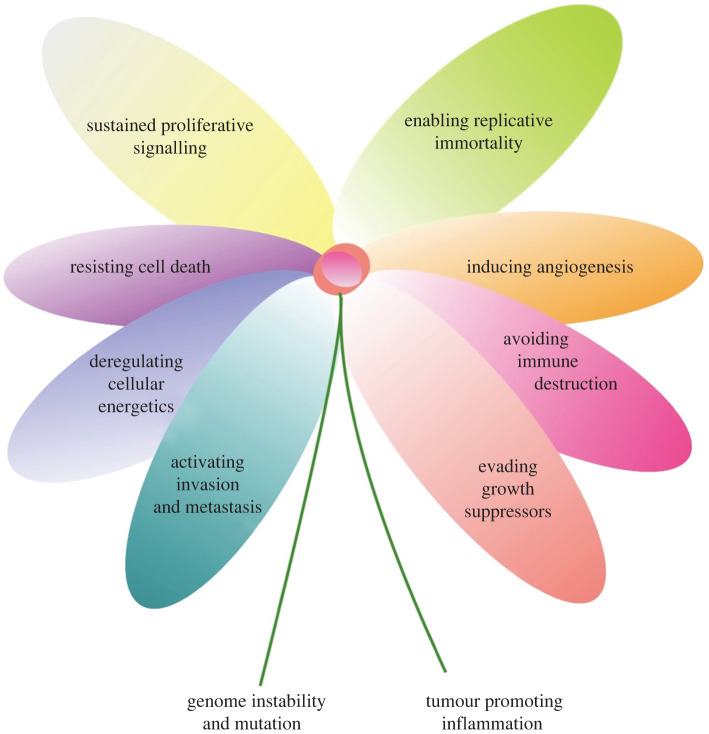
Hallmark flower.

Fast forward to 2000, Douglas Hanahan and Robert Weinberg compiled the key concepts surrounding cancer into the hallmarks of cancer, discussing the various mechanisms that underpin tumour development (figure 1).

## 2. Hallmarks of cancer

The challenges presented by multiple roadblocks, which are in place to prevent excessive cell proliferation and the development of tumours, leads to the daunting complexity of cancer. Tumour cells do not invent new mechanisms, but rather manipulate existing molecular and cellular pathways to circumvent protective mechanisms which are in place to prevent the formation of a tumour.

These conceptually distinct capabilities of tumour cells have a powerful resonance in the field of cancer therapeutics. Despite our knowledge of specific mutations in tumour cells generated through global sequencing efforts, such as the International Cancer Genome Consortium, the reductionist view would be to just focus on the cancer cell. However, we are actually dealing with a complex heterotypic tumour microenvironment where the tumour cells are only the foundation of cancer as a disease but not its complete manifestation.

**Figure 2.**
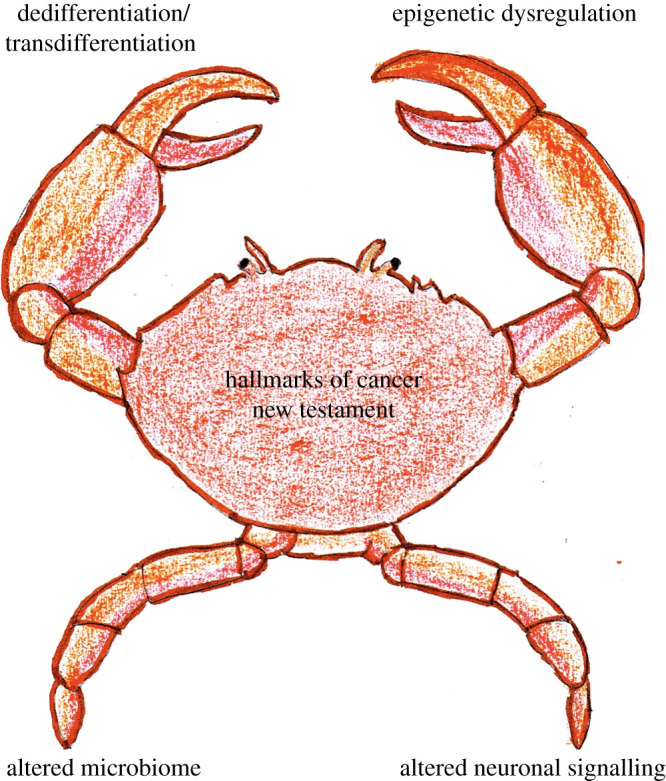
Novel hallmarks of cancer.

We propose four novel hallmarks of cancer, justify their importance in tumourigenesis and argue the need to incorporate them in the mainstream hallmark conceptualization (figure 2).

## 3. New hallmark 1: dedifferentiation and transdifferentiation

**Figure 3.**
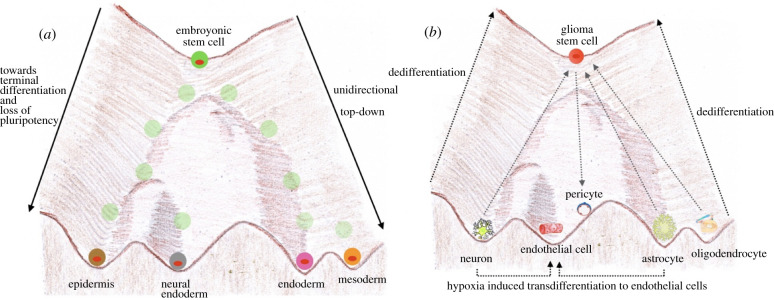
Tumour defiance of Waddington landscape. (*a*) Waddington landscape depicting the unidirectional nature of differentiation, adapted based on the concept of [8]. (*b*) The forgotten hallmark Dedifferentiation: Dedifferentiation from terminally differentiated neuron, astrocyte and oligodendrocyte, as well as transdifferentiation of neuron and astrocyte to endothelial cells.

In 1957, Conrad Waddington proposed the unidirectional developmental model, wherein pluripotent stem cells at the top of the hill progressively lose their pluripotency as they follow developmental pathways and end up among different valleys in a terminally differentiated state [7] (figure 3*a*). However, the concept of tumour cell plasticity goes against the Waddington landscape, where dedifferentiation allows non-cancer stem cells to acquire stem cell-like features.

In 1962, Sir John Gurdon challenged the unidirectional dogma of development with his ground-breaking study which showed the formation of a fully functional tadpole clone even when the nucleus of a frog zygote was replaced with a nucleus harvested from a terminally differentiated tadpole intestinal cell [9]. This proved his hypothesis that the genome of a mature specialized cell has all the information required to develop into the different cell types of an organism. However, Gurdon's experiment involved physical removal and transfer of cell nuclei and as such the question remained whether such a hypothesis could be replicated in intact cells. Forty years later, this question was answered with a proof of concept study which defied the Waddington landscape in intact cells. Introducing just four genes, the Yamanaka factors: *c-MYC*, *Kruppel-like factor 4* (*KLF4*), *Sex-determining region Y-box 2* (*Sox2*) and *Octamer-binding transcription factor 3/4* (*Oct-3/4*); Takashi and Yamanaka were able to develop what they termed induced pluripotent stem cells (iPSCs), which had the ability to differentiate into any of the cell lineages, endodermal, ectodermal and mesodermal [10]. This forms the basis of the hypothesis that tumour cells, which are champion survivors, will hijack any mechanism in order to survive; as such, dedifferentiation is a lucrative hallmark for them to achieve immortality.

Cancer stem cells (CSCs) are a unique subpopulation which possesses the cardinal property of self-renewal. This population can underpin tumour heterogeneity and resistance to cancer therapeutics, leading to relapse. The dedifferentiation of non-CSCs to CSC gives a survival advantage to cancers. Turning the clock back in time to a stem cell progenitor state is not a mere manifestation of the existing hallmark but a pivotal hallmark in itself and it further confers the ability to switch lineages, as lineage plasticity enables resistance against therapeutics. Let us consider some key examples that reiterate dedifferentiation as an integral hallmark of cancer.

### 3.1. Evidence of dedifferentiation in glioblastoma

The interconvertible nature of cancer stem cells and non-cancer stem cells can be seen within glioblastoma multiforme, a highly lethal sub-type of brain cancer. In 2002, a study reported that even mature astrocytes and neurons can be the cell of origin in certain brain tumours. EGFR activation and dual inactivation of p16^INK4a^ and p19^ARF^ cause astrocytes to undergo dedifferentiation to a multipotent progenitor state dictating the emergence of the high-grade phenotype of gliomas [11]. The extent of dedifferentiation of astrocytes is radical enough to give rise to pluripotent cells which have the ability to differentiate into glia as well as neurons, as evidenced by expression of neuronal marker TUJ1 among such tumours which arise from dedifferentiated astrocytes [11]. Indeed, the majority of mature differentiated cells in the central nervous system, given the right permissive microenvironment, can undergo dedifferentiation to a progenitor state, generating a neural stem cell that can perpetuate tumour progression as well as tumour heterogeneity and resistance to treatment [12] (figure 3*b*). Tumour plasticity allows for vascular mimicry via the transdifferentiation of glioblastoma cells into vascular endothelial cells [13] and even pericytes, which can support the maintenance of tumour vessel function [14] (figure 3*b*).

### 3.2. Evidence of dedifferentiation in intestinal tumours

Tumour initiating cells formed via dedifferentiation have also been reported in intestinal tumours. Enhanced NF-κB signalling leads to activation of β-catenin/TCF transcription via stabilization of β-catenin, inducing dedifferentiation of non-stem intestinal epithelial cells to intestinal epithelial cells with tumour initiating stem-like properties [15]. If Wnt activity plays a role in dedifferentiation of non-stem intestinal epithelial cells to tumour initiating cells, then a further investigation into whether this activity is mediated by the tumour microenvironment is warranted. In colon cancer, myofibroblasts in the tumour niche orchestrate high Wnt activity via β-catenin localization through hepatocyte growth factor secretion, which facilitates the reprogramming of the colon cancer cells to a stem cell-like progenitor state [16].

### 3.3. The pliability of cell fate in pancreatic cancer via dedifferentiation

There is a dynamic equilibrium between stem-like state and non-stem differentiated state. An activating mutation of the small GTPase KRAS is identified in about 90% of pancreatic tumours [17]. In a proof of concept study in pancreatic ductal adenocarcinoma, KRAS and its downstream target MYC were shown to rapidly reprogram differentiated mature cells to a stem cell-like state, poised to become malignant. Generation of metastatic pancreatic tumour cells with self-renewing capability is particularly shown to be controlled via MYC, which functions as a built-in amplifier [18].

Another study has shown that the major mechanism of initiation of pancreatic ductal adenocarcinoma lies in the synergism between the transcription factor SOX9 and activated KRAS, leading to the dedifferentiation of pancreatic acinar cells through a duct-like phenotype and the subsequent formation of pancreatic intraepithelial neoplasia [19]. Such genetic mutation induced dedifferentiation explains the differences in the kinetics of dedifferentiation of cells, at different states of differentiation in a tumour, which reiterates the need to target both cancer stem cells, and non-stem cancer cells to prevent re-initiation of tumourigenesis following therapy.

### 3.4. Therapy resistance via lineage plasticity through dedifferentiation

Despite achieving remission in metastatic melanoma with adoptive cell transfer therapies, there is frequent relapse. Relapse may be due to the secretion of the proinflammatory cytokine tumour necrosis factor (TNF)-α by T cells and macrophages in the tumour microenvironment, which results in reversible dedifferentiation of melanoma cells and thereby a loss of melanocytic antigens [20]. If dedifferentiation can aid in evasion from T cell immunotherapy, it raises the possibility of dedifferentiation as an enabling hallmark for immune evasion.

Dedifferentiation is also linked to resistance to targeted therapies in melanoma, for example, resistance to BRAF inhibition is conferred by the downregulation of microphthalmia-associated transcription factor (MITF), which plays a key role in melanocyte differentiation, and the upregulation of the receptor tyrosine kinase AXL, platelet-derived growth factor receptor and EGFR [21]. Dedifferentiation also provides cues for a potential susceptibility target, for example, the ability to induce ferroptosis, a form of iron-dependent necrotic cell death, in dedifferentiated melanoma cells [22]. As such, it can provide an option to induce a form of synthetic lethality by the combination of ferroptosis inducing drugs along with targeted therapies or immunotherapy in melanoma patients. Dedifferentiation-based changes have been found among melanoma patients even within the first week of targeted therapy treatment [23], suggesting a combination regimen with ferroptosis inducing drugs could possibly be initiated up-front to prevent escape via dedifferentiation.

Dedifferentiation has also been implicated in therapeutic resistance among prostate and breast cancers. A gain and loss of function study identified that upregulation of the reprogramming transcription factor Sox2 can confer reversible lineage plasticity by switching prostate cancer cells to a neuroendocrine phenotype, in the context of concomitant loss of tumour suppressors p53 and Rb [24]. Earlier studies showed that multipotent adipose-derived stem cells (ASCs), used in soft tissue reconstruction following mastectomy, undergo a phenotypic alteration via myofibroblastic differentiation, leading to contraction and enhanced stiffness, ultimately promoting tumourigenesis [25]. The impact of physical stresses on the tumour microenvironment leading to tumour progression has also been shown to involve triggering dedifferentiation. When a tumour grows, it causes compression of the surrounding tissue. Physical stress caused by mammary adenocarcinoma via compression of surrounding adipocytes triggers Wnt/β-catenin signalling and their subsequent dedifferentiation to myofibroblasts, which then interact with breast cancer cells leading to enhanced tumour proliferation [26].

### 3.5. Reflecting upon the Yamanaka factors' relationship to oncogenesis

A definitive proof of the importance of re-programming in cancer ontogeny is that each of the four Yamanaka factors capable of playing a role in dedifferentiation has an ascertained role in oncogenesis among multiple cancers. Oct4 is a biomarker for seminomas [27] and has also been attributed to the maintenance of the undifferentiated cell population with proliferative capacity by blockage of progenitor cell differentiation [28]. Sox2 is a key driver towards a stem cell fate among Ewing's sarcoma, breast and brain tumours [29,30]. Aberrant MYC expression has been strongly linked to several cancers [31] and KLF4 has been linked to colorectal cancer [32]. Though challenging targets themselves, Yamanaka factors may provide insight for the development of more targeted therapies.

The loss of *APC* maintains a progenitor state, following which oncogenic mutations such as KRAS can be acquired, driving tumourigenesis [33]. So, does dedifferentiation act as an enabling hallmark to grant time to acquire additional mutations to progress in the path towards tumour development? Or does dedifferentiation allow for lineage plasticity for tumour cells to alter their cell fate to a lineage more resistant to therapeutics?

In the case of acute promyelocytic leukaemia (APML), translocation results in promyelocytic leukaemia protein (PML) and retinoic acid receptor *α* (RAR*α*) fusion. The expression of the *PML-RARα* fusion gene blocks the terminal differentiation of granulocytes, resulting in the maintenance of neoplastic cells in the promyelocytic progenitor stage, but all-trans retinoic acid has been successful in overcoming the differentiation block by inducing differentiation of neoplastic cells into granulocytes [34]. The abrogation of terminal differentiation, as seen in APML, in order to maintain a progenitor-like state, supports the hypothesis of dedifferentiation as a logical hallmark. Even if cancer cells proceed to, or develop from, a state of terminal differentiation, they can revert back to their progenitor state and maintain their stemness via dedifferentiation. The Waddington landscape has been defied by cancer, providing tumour cells the plasticity to choose their fate by pushing the ball uphill against the landscape, to maintain cancer stem cells and underpin the basis of cancer as a lethal disease.

### 3.6. Therapeutic interventions based on the hallmark of dedifferentiation

**Figure 4.**
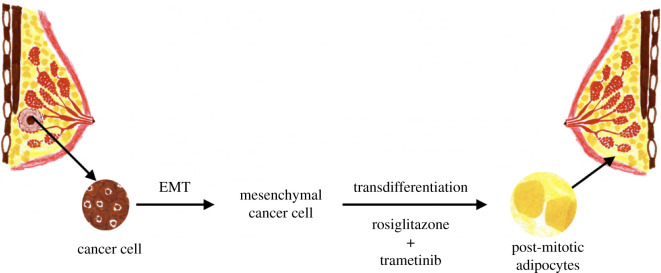
Transdifferentiation approach to therapy. Following combination of PPAR*γ* agonist rosiglitazone (an anti-diabetic drug) + MEK inhibitor—cancer cells are converted to functional adipocytes, adapted based on [38].

An important aspect of hallmarks of cancer conceptualisation is to aid in advancing therapeutic strategies, so an understanding of the nuances of the hallmark of dedifferentiation is important. There are three therapeutic modalities that can be targeted towards the tumour cell plasticity conferred by dedifferentiation:

1. Blocking dedifferentiation via combination therapies. Target the differentiated cell lineage along with drugs that block dedifferentiation in order to prevent early resistance to therapeutics as a result of lineage plasticity conferred by dedifferentiation.
2. Target dedifferentiation with differentiation therapy towards a permanently differentiated state. Initially attempted in the context of teratoma [35], but a proof of concept study for this approach was treating APML with all-trans retinoic acid therapy [34]. Other studies also reported a differentiation therapeutic approach aimed at the conversion of dedifferentiated tumour cells into epithelial cells that are more sensitive to chemotherapy [36,37].
3. Go with the flow and use the tumour plasticity to target the dedifferentiated cancer stem cells with transcription factors or small molecules to differentiate them into harmless cell lineages which lack tumourigenic potency. This final therapeutic approach necessitates an in-depth understanding of the hallmark of dedifferentiation. Recent work proved the efficacy of this approach by switching malignant breast cancer cells into harmless post-mitotic adipocytes. Combination of PPAR*γ* agonist Rosiglitazone, an anti-diabetic drug, with a MEK inhibitor was used to force the breast tumour cells towards adipogenesis, resulting in harmless post-mitotic functional adipocytes [38] (figure 4).

**Figure 5.**
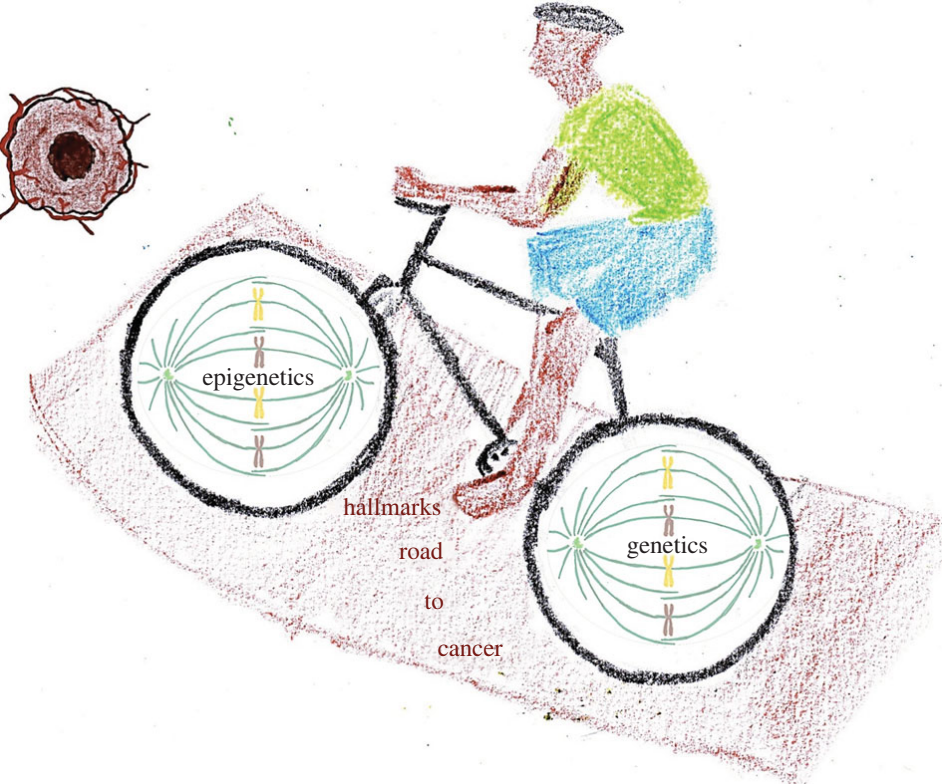
The road better not taken: intertwined nature of epigenetic instability and genetic instability in riding over the road of hallmarks towards tumourigenesis.

These studies argue strongly that the dedifferentiation of tumour cells along a developmental pathway towards a progenitor or stem cell-like state among various cancers is a forgotten hallmark, a discrete acquired capability of cancer and certainly warrants further investigation for a better understanding of this novel trait of cancer cells. Hanahan and Weinberg's Hallmarks of Cancer had generic nature as one of the features of every hallmark, as something that is prevalent in the majority of cancers despite the heterogenetic nature of the disease. Dedifferentiation certainly qualifies as a generic hallmark distinct from the other hallmarks of cancer. The reported interplay between transcription regulators Sox2 and Sox9 as an epigenetic switch between high proliferation and high invasiveness [39] leads to our next hallmark of cancer that influences tumourigenesis—epigenetic dysregulation (figure 5).

## 4. New hallmark 2: epigenetic dysregulation

Dedifferentiation to a progenitor state is a rate-limiting step in melanoma formation but it is underpinned by epigenetic machinery [40]. Although Yamanaka factors provide the possibility of reprogramming differentiated somatic cells to a pluripotent state, the blockage of histone H3 lysine 9 (H3K9) methylation has been shown to enhance this reprogramming capability [41,42]. Similarly, in the context of DNA methylation, another key epigenetic alteration, the promotion of DNA demethylation via stimulation of TET (ten-eleven-translocation) enzymes using vitamin C enhances reprogramming to a pluripotent state [43]. Epigenetics can also regulate the process of winding back the clock to a pluripotent state on the basis of chromatin state and the expression levels of chromatin-modifying enzymes [44], providing a conceptual link with our first hallmark of dedifferentiation.

### 4.1. What is epigenetics?

Among several phenomenal works, Theodor Boveri laid the foundation of the role of epigenetics in cancer through his observation of abnormal chromatin structures in tumour cells, described over 90 years ago [45]. The term ‘epigenetics’ was first coined by Conrad Waddington, defining it as ‘the branch of biology which studies the causal interactions between genes and their products which bring the phenotype into being' [8]. Vogelstein and Feinberg, in an attempt to dissect the mechanism underlying the higher frequency of mutations among tumours, compared normal tissue with tumour tissue and revealed the loss of DNA methylation in a substantial proportion of tumour tissues, positing that hypomethylation of CpG islands could lead to oncogene activation in cancer [46] and revealing the prevalence of global hypomethylation among tumour genomes.

Holliday refined the definition of epigenetics as heritable changes in the gene expression without alteration in the DNA sequence, that is altering the phenotype without altering the genotype [47]. With epigenetics playing a fundamental role in the development and progression of various cancers via modification of gene expression, such as hypermethylation of tumour suppressor genes in retinoblastoma [48] and epigenetic silencing of microRNAs [49], it is a pivotal hallmark of cancer.

### 4.2. Epigenetic fingerprints as the ‘midas touch' driving tumourigenesis—the hallmark of hallmarks

**Figure 6.**
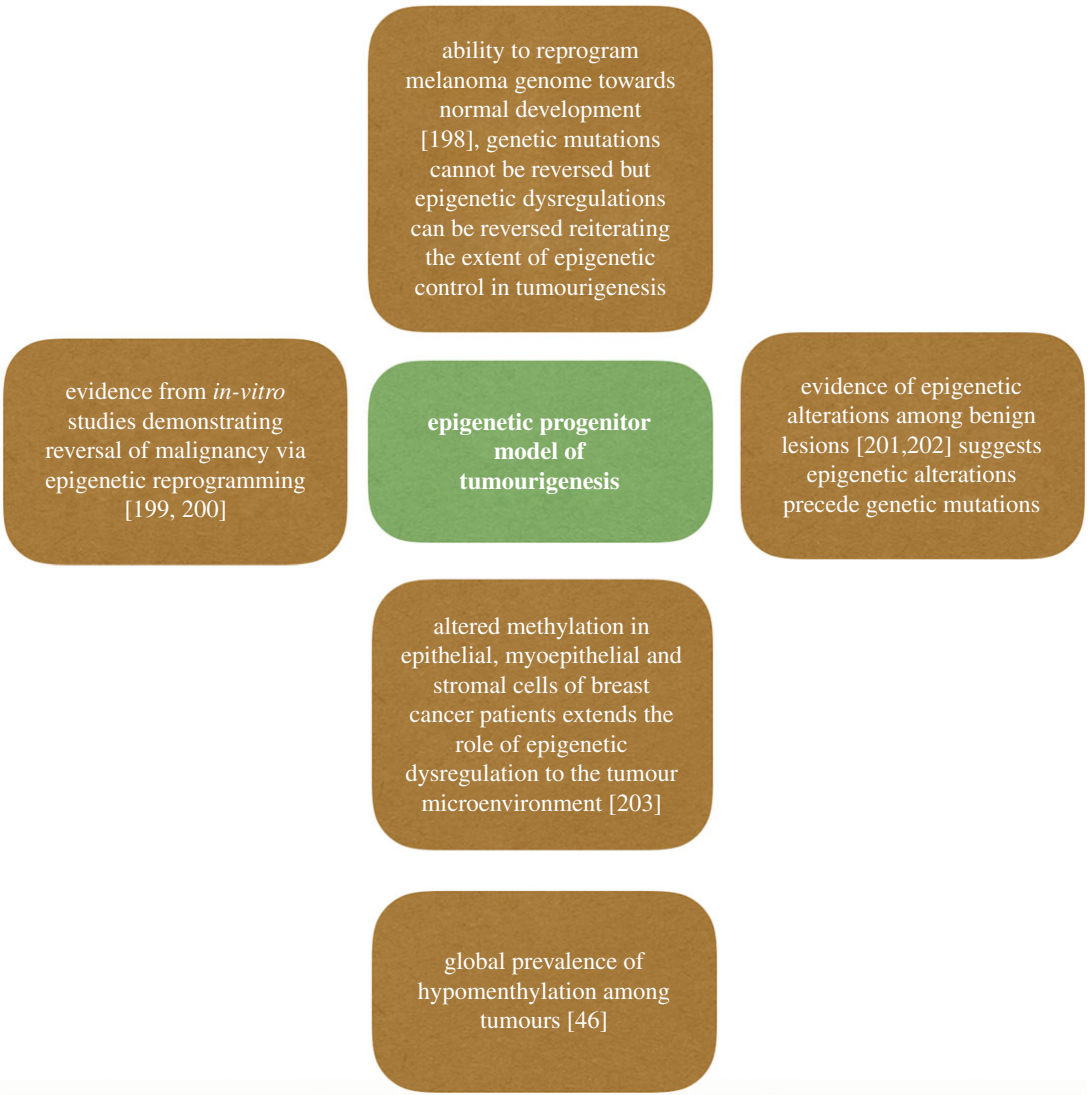
Epigenetic crossroads: multitude of studies supporting the epigenetic progenitor model.

As discussed in the features of a hallmark, epigenetic dysregulation is an active functional capability, it is a unique feature among cancer cells and there are epigenetic fingerprints on tumour cells which reflect its chronic nature. Epigenetic dysregulation viewed as a bystander would be a relegation of its active role in tumourigenesis, as several studies have pointed out its role in tumour initiation. In 2006, Feinberg proposed the epigenetic progenitor model of tumourigenesis, wherein epigenetic dysregulations of the progenitor cell population give rise to tumours [50] (figure 6).

### 4.3. Sustained proliferative signalling

Many tumours display a gain of function mutation of isocitrate dehydrogenase (IDH) [51,52], leading to the generation of the oncometabolite 2-hydroxyglutarate, which disrupts the function of hydroxylases such as TET — a key catalyst in the process of DNA demethylation [53,54]. The result is a hypermethylated phenotype as seen with the CpG island methylator phenotype (G-CIMP) in IDH mutant glioma [55]. This alters the binding affinity of the DNA binding protein CTCF (CCCTC-binding factor) which is very sensitive to methylation states [56]. CTCF has a critical function as an insulator, setting the boundaries which limit the interactions between an enhancer and a gene in the context of topologically associated domains (TADs) [56]. This insulation is lost as a result of the reduced binding of CTCF, facilitating aberrant interactions between promiscuous enhancers and genes as a result of the altered chromosomal topology caused by epigenetic dysregulation [57]. In this context, *Platelet-derived growth factor receptor A* (*PDGFRA),* a predominant oncogene among gliomas [58] is activated as a consequence of the epigenetic dysregulation-led decrease in CTCF insulation, with a potent promiscuous enhancer driving constitutive *PDGFRA* expression, driving sustained proliferation in gliomas [57]. The loss of CTCF insulation may even be preserved in subsequent cell divisions, compromising the genomic topology otherwise maintained by this insulation, leading to further oncogene activation not limited to just *PDGFRA* [59].

This mechanism is not limited to gliomas, as CTCF sites which are adjacent to oncogenes have been reported as mutational hotspots and are frequently mutated in multiple tumours, such as endometrial [60], colorectal (CRCs), oesophageal and liver cancer [59].

### 4.4. Evading growth suppressors

*Cyclin-dependent kinase inhibitor 2A (CDKN2A)* encodes a potent tumour suppressor p16^INK4a^, that binds to cyclin-dependent kinase 4/6 (CDK4/6), which leads to an allosteric conformational change inhibiting the cyclin D–CDK4/6 complex formation. As a result of the lack of this complex, retinoblastoma protein (Rb) is maintained in a hypophosphorylated state, promoting the formation of Rb/E2F repressive complex. This leads to the suppression of growth, as a result of cell cycle arrest in G1 [61]. Epigenetic silencing of tumour suppressors such as p16^INK4a^ via promoter hypermethylation mediates evasion of growth suppression, as evident from multiple studies on epigenetic alterations enumerated below (table 1).

**Table 1.** Epigenetic instability mediating evasion of growth suppressors.

type of cancer	frequency of promoter hypermethylation	reference
pancreatic adenocarcinoma	24.6%	[62]
oesophageal squamous cell carcinoma	81.7%	[41,42]
melanoma	25.9%	[63]
Burkitt's lymphoma	72.5%	[64]

Similarly, the hyperactivity of enhancer of Zeste homologue 2 (EZH2), a catalytic subunit of polycomb repressive complex 2 (PRC2) involved in the trimethylation of histone H3 lysine 27 to form H3K27me3, is implicated in the evasion of growth suppression via *CDKN2A* repression [65–67] reiterating the role of epigenetic dysregulation in facilitating the hallmarks [62].

### 4.5. Invasion and metastasis

An integral component of the hallmark of invasion and metastasis is a reversible epithelial–mesenchymal transition (EMT), orchestrated by the interaction between epigenetic modulators of chromatin configuration and EMT inducing transcription factors. Expression of E-cadherin, a key coordinator of epithelial phenotype, is lost during EMT. Epigenetic repression of *CDH1,* which encodes E-cadherin, is mediated by the recruitment of EMT inducing transcription factor Snail to the *CDH1* promoter, leading to a repressive mark H3K27me3 [68]. Further to this, Snail can associate with Mi-2**-**nucleosome remodelling and deacetylase (NuRD) repressive complex, which can repress *CDH1* activity via deacetylation of *CDH1* promoter [69].

### 4.6. Replicative immortality

Alternative telomere lengthening (ALT) is a telomerase-independent homologous recombination-based pathway which cancer cells use to overcome the Hayflick limit to maintain telomere length [70]. An interplay between epigenetics and genetic mutations leads to perturbations of histone variant H3.3 and its specific chaperone proteins α-thalassemia X-linked mental retardation protein (ATRX) and death domain associated protein (Daxx) which impairs the incorporation of the histone variant H3.3 at telomeres, disrupting their heterochromatic state and facilitating ALT [71].

### 4.7. Inducing angiogenesis

Epigenetics plays a key role in angiogenesis. Histone deacetylases have been shown to downregulate the expression of von Hippel–Lindau (VHL) and p53, but promote an increase in hypoxia-inducible factor-1*α* and vascular endothelial growth factor (VEGF), thereby stimulating angiogenesis by the suppression of hypoxia-responsive tumour suppressor genes [72,73]. Choriocarcinoma, a highly vascular tumour derived from trophoblasts, displays epigenetic silencing of *FLT1* via promoter hypermethylation. Normal placental trophoblasts express abundant levels of an anti-angiogenic factor, Soluble Fms-like tyrosine kinase-1 (sFLT1) from the *FLT1* locus. Epigenetic silencing of *FLT1* blocks expression of this negative regulator, thereby facilitating angiogenesis in choriocarcinoma [74].

### 4.8. Resisting cell death

Glioblastoma multiforme is a very aggressive cancer with a dismal prognosis. Nevertheless, a promising therapeutic strategy is to induce tumour cell death via tumour necrosis factor-related apoptosis-inducing ligand (TRAIL)-based therapy that binds to human death receptor 4 (DR4). However, epigenetic silencing via promoter methylation of *DR4* attenuates TRAIL/DR4-mediated apoptosis [75]. Further evidence of epigenetics mediating resistance to cell death is resistance to anthracycline therapy in acute myeloid leukaemia (AML), due to impaired DNA damage response from defective nucleosome remodelling, as a result of mutation of epigenetic regulator DNA methyltransferase 3A [76]. CXCL14, a chemokine which can influence apoptosis, is shown to be a frequent candidate for epigenetic silencing among lung tumours. Tumour specific methylation of the CXC-subfamily of chemokines was observed in 75% of lung adenocarcinomas [77].

### 4.9. Immune evasion

Epigenetics is fundamental to the normal functioning of immune cells. Antigen presentation through MHC class I is pivotal for CD8+ T cells activity. The class I transactivator *NLRC5* is a transcriptional regulator of the MHC class I genes, but the promoter region of *NLRC5* is frequently methylated among cancers, resulting in the reduction of MHC class I gene expression [78].

### 4.10. Deregulating cellular energetics

In order to adapt to a hostile microenvironment and to satisfy their high metabolic needs, cancer cells can use glycolysis, instead of oxidative phosphorylation to metabolize glucose, even in aerobic conditions. The central activators implicated in the glycolytic phenotype are the PI3 K/AKT/mTOR pathway along with MYC and HIF-1 signalling [79].

Tumour suppressors that repress this pathway, namely *PTEN* [80], *VHL* [81,82], *LKB1* [83] and prolyl hydroxylases [84] are epigenetically silenced via promoter hypermethylation, contributing to deregulation of cellular energetics.

### 4.11. Genomic instability and mutation

Faithful genome replication and the maintenance of genomic integrity are underpinned by epigenetic mechanisms. Transposable elements (TE) are highly repetitive sequences of DNA in the human genome, and have their own regulatory sequence, allowing for independent expression and ability to alter the expression of neighbouring genes. Since TE activity has a high propensity to disrupt genomic integrity, these are usually silenced epigenetically, but this regulation is lost in cancer [85].

### 4.12. Tumour promoting inflammation

DNA demethylation triggers transcription of inflammation-related genes, including *chemokine receptor 4* (*CXCR4*) and *serum amyloid A* (*SAA*) in advanced clear cell renal cell carcinoma (ccRCC), contributing to tumour promoting cancer cell-intrinsic inflammation via epigenetic remodelling [86].

The above studies highlight an epigenetic foundation for each of the established hallmarks of cancer provides compelling evidence of the indispensable nature of epigenetic dysregulation as a pivotal enabling hallmark of cancer. However, just as cancer involves more than just tumour cells, so our bodies are more than simply an assemblage of human cells. This sobering thought leads us to our third hallmark, the microbiome.

## 5. New hallmark 3: altered microbiome

**Figure 7.**
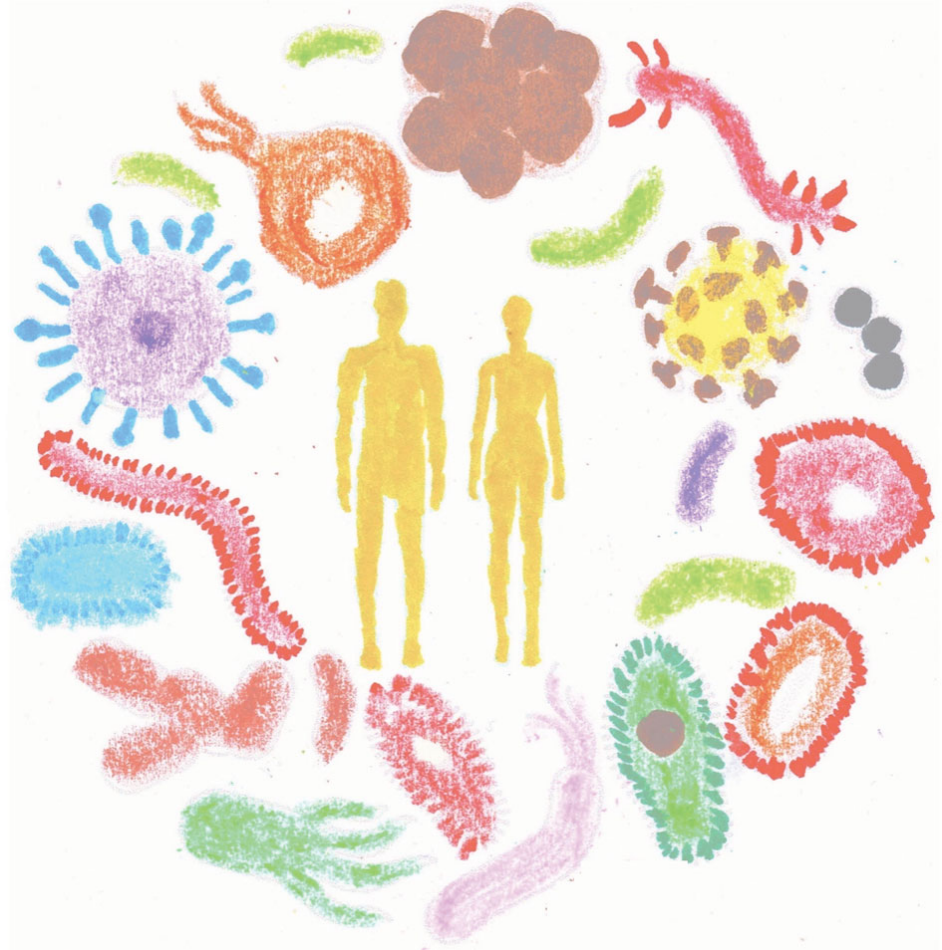
Coexistence.

The concept of the human body being a vessel for other microorganisms is well established—the microbial metagenome in our body outnumbers our genome by at least 100-fold [87]. Microorganisms first appeared around 3.25 billion years ago [88] and over the 1.25 billion years of co-existence with multicellular eukaryotes [89] (figure 7), the interaction with microbes has shaped evolution, as illustrated by the microbial control of host homeostasis [90]. It has been estimated that nearly half of the metabolites in plasma are of microbiotal origin [91], but the human microbiome plays a duplicitous role. *Helicobacter pylori* is nearly ubiquitous among humans, colonizing about 50% of the world population, having co-evolved with humans in an association spanning over 50 000 years [92]. *H. pylori* colonization has been shown to decrease the risk of gastroesophageal reflux disease and its subsequent sequela, oesophageal carcinoma [93]. It may also confer protection against asthma [94], demyelinating diseases such as multiple sclerosis [95], tuberculosis [96] and inflammatory bowel disease [97]. *H. pylori* also has been shown to modulate energy homeostasis via cooperation with gut microbiota, impacting on circulating metabolic gut hormones [98].

In contrast with these beneficial roles, as a component of the gut microbiome it is also linked to 90% of gastric cancers [99]. The carcinogenicity of *H. pylori* is associated with the expression of *vacuolating cytotoxin gene A* (*vacA*) and *cytotoxin-associated gene A* (*CagA*) [100,101]. *CagA* positive *H. pylori* promotes genetic instability via perturbation of the mitotic spindle checkpoint, causing chromosomal instability [102] and epigenetic instability. Increased levels of DNA methyltransferases (DNMTs) [103] lead to hypermethylation of *MLH1,* a key DNA mismatch repair gene [104], and have been suggested to mediate a mutator phenotype in a hit and run fashion, promoting tumourigenesis [101]. Given these data, it is worthwhile to postulate whether *Escherichia coli* has a role in cancer, being among the first bacteria to colonize the gastrointestinal tract of neonates [105] and advocated to promote gut health in multiple off the shelf probiotics [106].

Commensal *E. coli*, within few days following birth, establish a favourable anaerobic environment in the gut facilitating colonization of other species including *Bifidobacterium*, *Clostridium* and *Bacteroides* [107]. Certain strains of *E. coli* harbour a gene cluster hybrid non-ribosomal peptide synthetase-polyketide synthase (*pks*) island which produces genotoxic colibactin [108]. Colibactin has been described as a bacterial ‘warhead', forming bulky unstable DNA adducts via alkylation [109]. Among epithelial cell lines, *pks^+^ E. coli* has been shown to induce double-stranded DNA breaks [110] and interstrand cross-links [111].

Whole-genome sequencing studies have described mutational signatures from colorectal crypts of healthy individuals where, in a subset of crypts, an unknown mutagenic agent caused co-occurrence of single-base substitution-A (SBS-A) and insertion/deletion A (ID-A). The two motifs were described to be from mutagenic insult which occurs in early childhood [112]. The cause of these two motifs surfaced while investigating the long-term effect of colibactin using single-cell-derived organoids, with *pks*-mutational signature strongly matching the two motifs SBS-A and ID-A [113]. Since *pks^+^ E. coli* is the mutagenic agent responsible, and the study was performed on organoids that cannot precisely mimic inflammation or the immune environment, the inference is that colibactin can directly initiate tumourigenesis via mutations [113]. Interestingly, the impact of these data reaches beyond the gut, suggesting a similar role in head and neck as well as urogenital cancers [113]. So, perhaps one should reconsider probiotics containing genotoxic *E.coli* and consider screening for *pks^+^ E. coli* in the context of colorectal cancer prevention.

Arguing for a microbiotal impact merely on the hallmark of genomic instability as being sufficient for its contribution to tumourigenesis is to grossly underestimate the role of the microbiome. Paget's seed (cancer cells) and soil (tumour microenvironment) hypothesis [114] is highly relevant to the role of the microbiome in tumourigenesis. The microbiome can exploit the inflammatory milieu to a pro- or anti-tumour state, cultivating the soil which is apt for sowing the seeds of tumourigenesis. This can be clearly substantiated by findings from a study using the first identified oncovirus [115], where Rous sarcoma virus does not induce tumours in sterile embryos despite expression of the *v-Src* oncogene [116].

In 1990, Fearon & Vogelstein [117] proposed the Vogelgram model of multi-step colon cancer pathogenesis. A key reason for the success of colon screening in CRC prevention is due to the long latency period from tumour initiation to overt clinically detectable CRC. Here, we consider this long latency in the context of the proposed hallmark of microbiome dysbiosis.

### 5.1. Microbiome tug-of-war hypothesis

**Figure 8.**
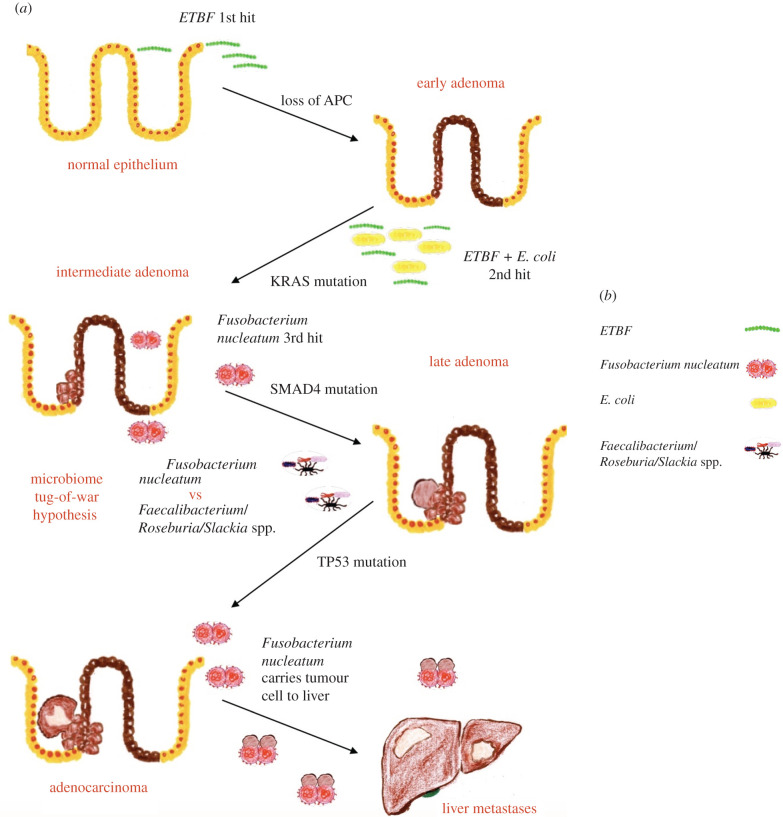
(*a*,*b*) Microbiome tug-of-war hypothesis for CRC latency: improvization of the Vogelgram [117], with an explanation of the role of bacteria in multi-step CRC progression.

The tug-of-war between microbiome species may underlie the long latency in CRC. Enterotoxigenic *Bacteroides fragilis* (ETBF) promotes the colonization of *pks^+^ E. coli*, together with leading to genetic and epigenetic instability. Following this, colonization by pro-tumourigenic *Fusobacterium nucleatum* further promotes tumourigenesis by aiding in the development of an immunosuppressive microenvironment and seeding metastasis, whereas anti-tumourigenic bacteria act to prevent malignancy. The long latency period, which may ultimately lead to subsequent accumulation of genetic/epigenetic mutations and overt malignancy, depends on the balance between pro/anti-tumourigenic microbes (figure 8).

ETBF secretes a 20 kDa zinc-dependent metalloprotease toxin, *B. fragilis* toxin (BFT). BFT degrades E-cadherin, leading to increased intestinal epithelial cell proliferation and permeability of the intestinal barrier [118]. BFT further leads to activation of β-catenin signalling and induces STAT3 (signal transducer and activator of transcription 3) activation [119] and the T helper 17 (TH17) immune response [120]. ETBF modulates the colonic niche to select for bacteria with a colonization advantage, inducing upregulation of antimicrobial peptide lipocalin 2 [121] that causes sequestration of bacterial siderophores. Siderophores are iron-binding complexes that are pivotal for bacteria to thrive in iron limiting environments, hence bacteria which are resilient to lipocalin 2, such as *E. coli*, begin to thrive along with ETBF [122]. Therefore, the first hit in our hypothesis of CRC tumourigenesis is orchestrated by ETBF followed by the co-colonization of ETBF along with *pks*^+^ *E. coli*, following which *Fusobacterium nucleatum* comes into play.

An anaerobic Gram-negative bacterium, *Fusobacterium nucleatum* is typically resident in the oropharynx, participating in dental biofilm formation [123]. Its virulence factor FadA adhesin binds to the extracellular domain of E-cadherin and promotes tumourigenesis via β-catenin/Wnt signalling [124]. *F. nucleatum* is also immunosuppressive, causing inhibition of T cell responses while allowing for the expansion of tumour promoting myeloid-derived immune cells [125]. Fap2 protein of *F. nucleatum* binds directly to the inhibitory receptor—T cell immunoglobulin and ITIM domain (TIGIT), and inhibits natural killer (NK) cell activity, leading to immune evasion [126]. In support of this hypothesis, *F. nucleatum* did not initiate tumour formation *in vivo* [127], but sustained the pro-tumourigeneic momentum in the latter part of multi-step CRC tumourigenesis and facilitated metastasis [128]. Intriguingly, a study based on biopsies from CRC patients and mouse xenografts revealed that *F. nucleatum* can accompany primary colorectal adenocarcinoma cells to distant metastatic sites, being maintained among patient-derived xenografts of CRC even through multiple passages. Moreover, treatment with metronidazole, an antibiotic to reduce *F. nucleatum* load, resulted in reduced tumour growth [128], suggesting that tumour cells are rewarded for carrying *F. nucleatum* by its modulation of the microenvironment at the distant metastatic site in favour of tumour growth.

Meanwhile, there is a subset of anti-carcinogenic bacteria including *Faecalibacterium*, *Roseburia* and *Slackia* spp. which generate catabolites such as short-chain fatty acids (SCFAs), for example, butyrate [129] and the antioxidant equol [130]. Butyrate downregulates proinflammatory gene expression and suppresses tumour growth via inhibition of histone deacetylases [131]. The outcome of the tug-of-war depends on epigenetic factors such as diet which will give the final upper hand aiding in colonization by either pro- or anti-carcinogenic bacteria.

The majority of pancreatic ductal adenocarcinoma (PDAC) patients have a dismal prognosis, but a small subset of patients survive longer than 5 years [132]. Intriguingly, long-term survivors have higher tumour microbial diversity with distinct tumour microbial signatures compared to short-term survivors [133]. The tumour microbial diversity was shown to exert an immune-activating effect via improved immune cell infiltration to the tumour milieu. Furthermore, colonization of pancreatic tumours by gut microbiota was identified, with 25% of PDAC microbial composition matching that of the gut. Preclinical data from the same study showed that faecal microbial transplant (FMT), from patients who were long-term survivors, into tumour-bearing mice led to immunoactivation in the murine tumour microenvironment and a significant reduction in tumour growth, reiterating the role of tumour microbiome in disease progression and outcome, as well as the potential of FMT in treating PDAC [133].

### 5.2. The microbiome is more than bacteria

**Figure 9.**
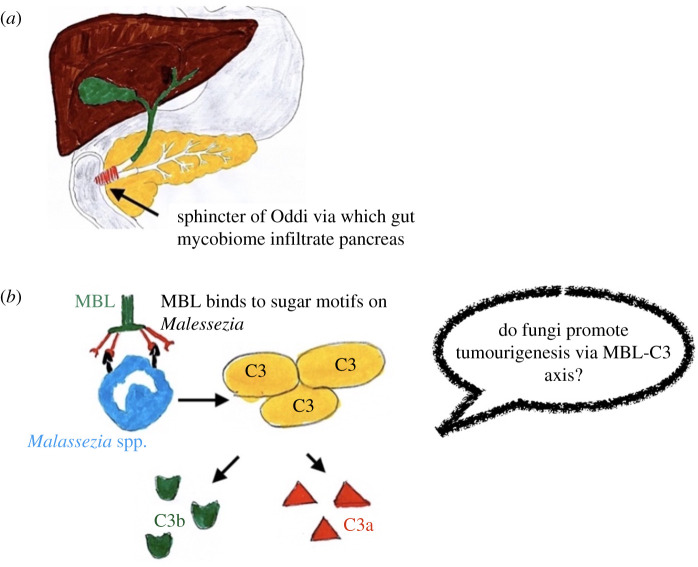
Tumour promoting inflammation triggered by mycobiome. (*a*) Sphincter of Oddi. (*b*) The mannose-binding lectin (MBL) pathway of complement activation, adapted based on [134].

We need to consider more than simply bacteria and viruses. Fungal infiltration from the gut to the pancreas was shown to occur via the sphincter of Oddi (figure 9*a*), which serves as a direct link between the pancreatic duct and the gut. Taxonomic diversity analysis identified the dominance of the genus *Malassezia* in PDAC tissues compared to that of the gut, in mouse models. Comparison of sequencing data of PDAC patient faecal samples to that of paired tumour tissue corroborated these findings. Antifungal ablation with amphotericin B mitigated pancreatic dysplasia in mouse models and was shown to work synergistically with gemcitabine in reducing tumour burden [135]. Through repopulation experiments, *Malassezia globosa* was identified as being responsible for PDAC disease progression, via fungal-mediated activation of the mannose-binding lectin (MBL)–C3 cascade (figure 9*b*). MBL is a protein of the innate immune system which serves as an opsonin. Upon binding to the sugar motifs on the fungal wall, it triggers the complement cascade, in particular C3, a pivotal component downstream of MBL [135]. Based on the inference from the study, we can speculate that diagnostic assay using the taxonomic composition of stool samples may be appropriate for early detection of PDAC, and that antifungal therapy may be efficacious.

More than 85% of human papillomaviruses (HPV) are cleared spontaneously [136], so why can the remaining 15% mediate progression to cervical neoplasia? The answer lies with the vaginal microbiome, dysbiosis of which plays an important role even in HPV-related cervical cancers [137]. *Lactobacillus* species are dominant in the vaginal niche and are characteristic of vaginal health [138]. They maintain the vaginal microenvironment in an acidic state (pH < 4.5) via the production of lactic acid [138] and protect against invading pathogens such as herpes simplex virus [139], human immunodeficiency virus [140], *Neisseria gonorrhoeae* [141] and even *E. coli* [142]. The depletion of *Lactobacillus* species has been linked to an increased risk of acquisition of HPV infection and its reduced clearance [137], and reduction in *Lactobacillus* dominance and increased vaginal microbiome diversity correlated strongly with cervical neoplasia severity [137,143].

The microbiome also plays an important role in deciding the outcome of both conventional chemotherapies and immunotherapeutic interventions. It can alter the bioavailability of drugs [144], and DNA damage induced by platinum-based regimens is severely attenuated in the absence of commensal microbiota [145]. Oral administration of *Bifidobacterium* in mice controlled melanoma growth on a par with checkpoint blockade using programmed cell death ligand 1 (PD-L1) specific antibody and co-administration resulted in near eradication of tumour growth [146]. Furthermore, the efficacy of blocking CTLA-4, a major negative regulator of T cell activation, depends on *Bacteroides* species and tumours in axenic or antibiotic-treated mice do not respond to CTLA-4 blockade [147]. The microbiome also has a role in immunosurveillance, as seen with the hygiene hypothesis which links an increase in the incidence of some cancers to decrease in exposure to certain microbes [148,149].

In conclusion, the microbiome exerts both beneficial and nefarious effects over the human body. We argue that it has a role in each of the triumvirate of immunoediting [150], namely elimination, equilibrium and escape during tumourigenesis and as such is a pivotal enabling hallmark of cancer. Antibiotic mediated alteration of gut microbiota has been shown to alter the cerebral tumour microenvironment, thus affecting glioma progression [151], which bring us to our final enabling hallmark of cancer—nerves/neuronal signalling.

## 6. New hallmark 4: altered neuronal signalling

**Figure 10.**
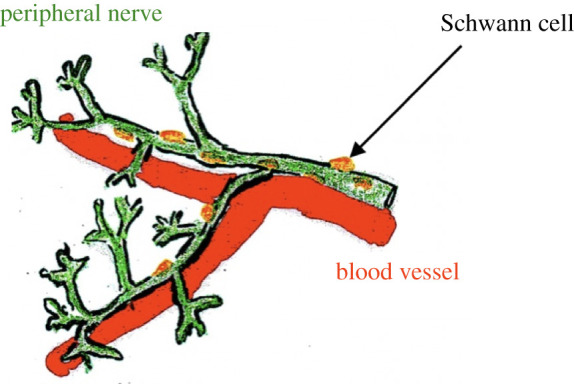
Blood vessels and nerves in tandem.

Vesalius, in his book *De corporis humani fabrica libri septem*, described the tandem nature of blood vessels and nerves [4,198] (figure 10), innervation and blood supply being indispensable for growth and survival. Since angiogenesis has an established role, it is enticing to delve further into the role of nerves in cancer, a topic that is often overlooked. Perhaps the reason might be the difficulty involved in observing nerves during routine histology of tumour specimens, but nerves are one of the most significant aspects of tumour progression. Metastasis involving the central nervous system/peripheral nervous system results in manifold increased morbidity/mortality.

In 1840, surgeons attempted to transect the trigeminal nerve, which runs along the face, and the accompanying blood vessels, in order to cure tumour of the lips. It provided symptomatic control but failed to cure the patients and eventually mandated complete resection of the tumour [152]. However, with recent advances in the understanding of the function of the nervous system, its role in tumour initiation and progression can be better elucidated, to derive therapeutic benefits. The density of nerve fibres in tumour tissue correlates with the aggressiveness of the disease among multiple cancers, including breast [153], lung [154], colorectal [155] and prostate cancers [156]. Based on these observations, one might advocate neuronal transection to control tumour progression. However, in a PDAC mouse model, sub-diaphragmatic vagotomy, targeting the vagus nerve—a mixed nerve with both sensory and parasympathetic components, resulted in enhanced tumour growth and reduction in survival [157]. Contrastingly, transection of the same nerve in gastric cancer models resulted in suppression of tumourigenesis [158]. Instead of the radical approach of transection, an alternative approach is to use chemical denervation, as performed in specific targeting of the sensory nerves in ductal carcinoma, and the use of capsaicin inhibited pancreatic ductal adenocarcinoma (PDAC) progression [159]. Another approach is via injection of botulinum toxin A (Botox), a neurotoxin, into the gastric wall. This inhibited progression to overt adenocarcinoma among preneoplastic models and inhibited disease progression in advanced gastric cancer models [158].

### 6.1. β-blockers for inhibiting tumour progression

Sympathetic nerves are implicated in blood vessel patterning during early development [160]. Sympathetic nerves release noradrenaline, the circulating levels of which increase during chronic stress [161]. β-adrenergic receptors mediate most of the effects of noradrenaline. Chronic stress has long been attributed as a risk factor for cancer [162]. Reproduction of the effect of chronic stress in transgenic mouse models of breast cancer via long-term administration of isoprenaline, a non-selective β-adrenergic agonist, resulted in increased lymph node metastasis, while inhibition of adrenergic signalling with propranolol, a non-selective β-blocker, resulted in inhibition of metastasis to the lymph node [163]. Reiterating the role of β-adrenergic receptors, a similar effect was also observed in pancreatic cancer models of chronic stress, with a reduction of tumour volume following administration of propranolol [164].

The pro-tumourigenic effects exerted by sympathetic nerves in response to stress are mediated by β-adrenergic receptors. This was demonstrated elegantly in work showing that an adrenergic nerve-derived signal-mediated activation of an angiogenic switch in a transgenic mouse model of prostate cancer [165].

Sympathetic nerves in prostate tumours release noradrenaline which, via the β2-adrenergic receptor on endothelial cells, triggers an angiogenic switch by inducing a change in endothelial cell metabolism from oxidative phosphorylation towards aerobic glycolysis, driving angiogenesis and fuelling tumour progression. Blockade of β-adrenergic receptor signalling reverts endothelial cell metabolism from aerobic glycolysis towards oxidative phosphorylation through cytochrome C oxidase assembly factor 6 (Coa6) activity, thereby inhibiting angiogenesis and curtailing tumour progression [165].

### 6.2. Perineural invasion in pancreatic ductal adenocarcinoma

**Figure 11.**
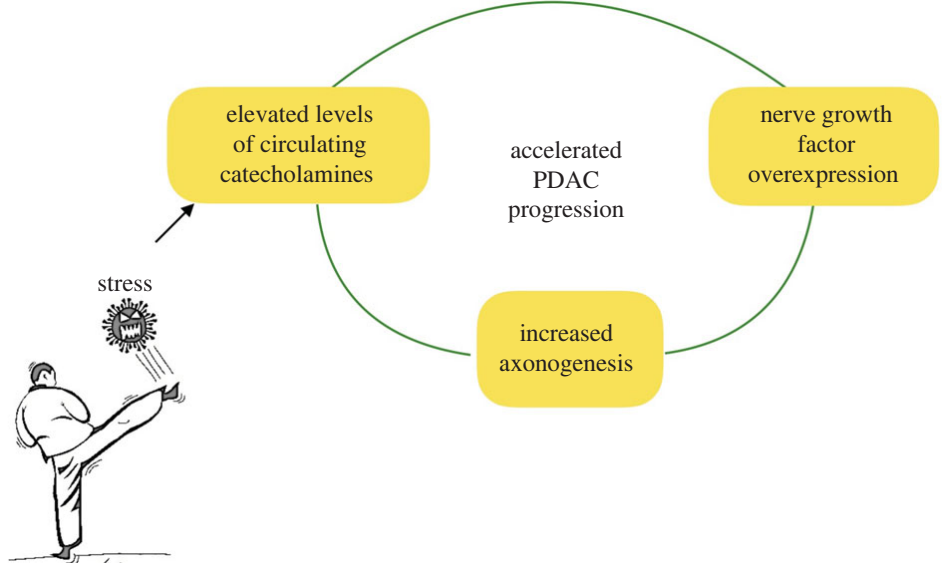
Chronic stress-dependent sympathetic nerve signalling triggering tumour growth via a feedforward loop.

Perineural invasion is linked to worse prognosis in PDAC [166], with PDAC cells recruiting nerves via nerve growth factor (NGF) [167]. In murine PDAC models, chronic stress-dependent sympathetic nerve signalling triggers tumour growth via a feedforward loop, wherein adrenergic signalling stimulates NGF, which promotes further innervation of tumour cells via axogenesis, resulting in increased noradrenaline accumulation in the tumour microenvironment, inducing β2-adrenergic receptor-dependent PDAC progression [168] (figure 11).

Blockade of the β2-adrenergic receptor, or the NGF receptor tropomyosin-receptor kinase A (TRKA), disrupts this feedforward loop and inhibits tumour progression [167,168]. Clinical studies have reported improved survival among PDAC patients with the use of β-blocker [169]. This provides a window of opportunity to treat patients with pancreatic intraepithelial neoplasias (PanINs) with a non-selective β-blocker regimen to potentially prevent progression to overt PDAC, although the challenge of early detection remains. This might be facilitated using a diagnostic assay based on the taxonomic composition of stool samples, as discussed earlier.

While β-adrenergic signalling is pro-tumourigenic in the aforementioned solid cancers, there is the caveat of an opposite effect adding to the complexity of targeting the hallmark of nerves/neuronal signalling. Noradrenaline-mediated sympathetic nerve signalling has been linked to the maintenance of the steady-state condition of haematopoietic stem cells (HSCs) in the bone marrow niche in a circadian manner [170]. Attrition of the β-adrenergic signalling leads to an increased propensity of myeloproliferative neoplasms [171,172], therefore the implementation of β-blocker targeting sympathetic signalling in malignancy is context-dependent.

### 6.3. Putting the cart before the horse: whether nerves migrate towards tumours or the tumour cells migrate towards nerves?

Schwann cells, the glial cells responsible for myelinating peripheral nerves, are key to neural homeostasis, participating in Wallerian degeneration, neural repair and regeneration [173]. In an *ex vivo* model using rat sciatic nerve, Schwann cells displayed a high affinity towards pancreatic and colon tumour cells, but not normal cells, migrating towards tumour cells, thereby outlining a pathway for tumour driven neurogenesis [174]. Nerve growth factor (NGF), and its receptors TRKA and p75NTR are critical regulators of gland innervation and neurite outgrowth. They are also implicated in neural tracking [175], the ability of tumour cells to migrate along axons.

Pro-NGF, the precursor of NGF, serves as a reservoir for NGF [176]. Immunohistochemical studies in prostate cancer suggested that pro-NGF production by tumour cells might drive axonogenesis [177]. Thus, there is an element of reciprocal interaction between nerves and tumour cells driving tumourigenesis; Schwann cells migrate towards tumour cells while prostate tumour cells in turn recruit nerves via pro-NGF.

### 6.4. Synaptic interaction between brain tumour cells and neurons

**Figure 12.**
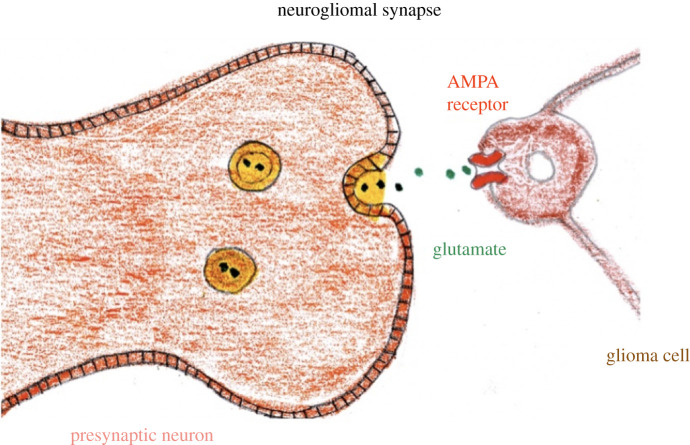
Synaptic interaction between presynaptic neuron and glioma via AMPA receptor.

Clues to the interaction between tumour cells and neurons come from the study of synapses between neurons and oligodendrocyte precursor cells, demonstrating a neuron to non-neuron synapse [178], as well as the finding that glutamate secretion confers a growth advantage to glioma cells [179]. These preliminary studies were bolstered by the identification of functional synapses between neurons and glioma cells, with transcriptomic analysis further confirming that the glioma cells express GluA2, a subunit of the ionotropic glutamate receptor, α-amino-3-hydroxy-5-methyl-4-isoxazole propionic acid receptor (AMPAR). Treatment with an AMPAR antagonist inhibited glioma progression, suggesting glioma cells can co-opt glutamatergic signalling to facilitate invasion and tumour progression [180,181] (figure 12). Based on both of these studies, one can speculate whether anti-epileptic drugs that act presynaptically, such as levetiracetam [182], might inhibit glioma progression. An alternative approach could be a non-competitive AMPAR antagonist such as perampanel, which has good penetration to the brain, for use in glioma treatment [183]. Key to note is that the AMPARs mentioned in both the studies [180,181] are calcium permeable, which means the target of the drug candidate must be calcium permeable AMPAR

Metastasis to the brain presents a checkmate scenario to clinicians but a breakthrough finding, deciphering the interaction between neurons and metastatic cells [184], can now pave way for new therapeutic approaches.

### 6.5. Parasitic tripartite synapse

Triple-negative breast cancer (TNBC) carries a poor prognosis as it lacks the expression of targetable hormone receptors and human epidermal growth factor receptor 2, coupled with a propensity to metastasize to the brain [185]. *N*-Methyl-d-aspartate receptor (NMDAR), a type of glutamate receptor, plays a key role in the synaptic plasticity of the central nervous system, but has also been implicated in ovarian and pancreatic tumour progression [186]. Transcriptomic data identified higher expression of NMDAR among basal sub-types of breast cancers such as (TNBC), in particular the NMDAR GluN2B subunit, which contains phosphorylation sites critical for NMDAR signalling. An autocrine source of glutamate-mediated NMDAR signalling in the breast to brain metastasis (B2BM) microenvironment was excluded, with B2BM cells found to express neuroligin-2 [184], the expression of which by non-neuronal cells has been shown to induce presynaptic differentiation and trigger *de novo* formation of pseudo-synapses [187,188].

**Figure 13.**
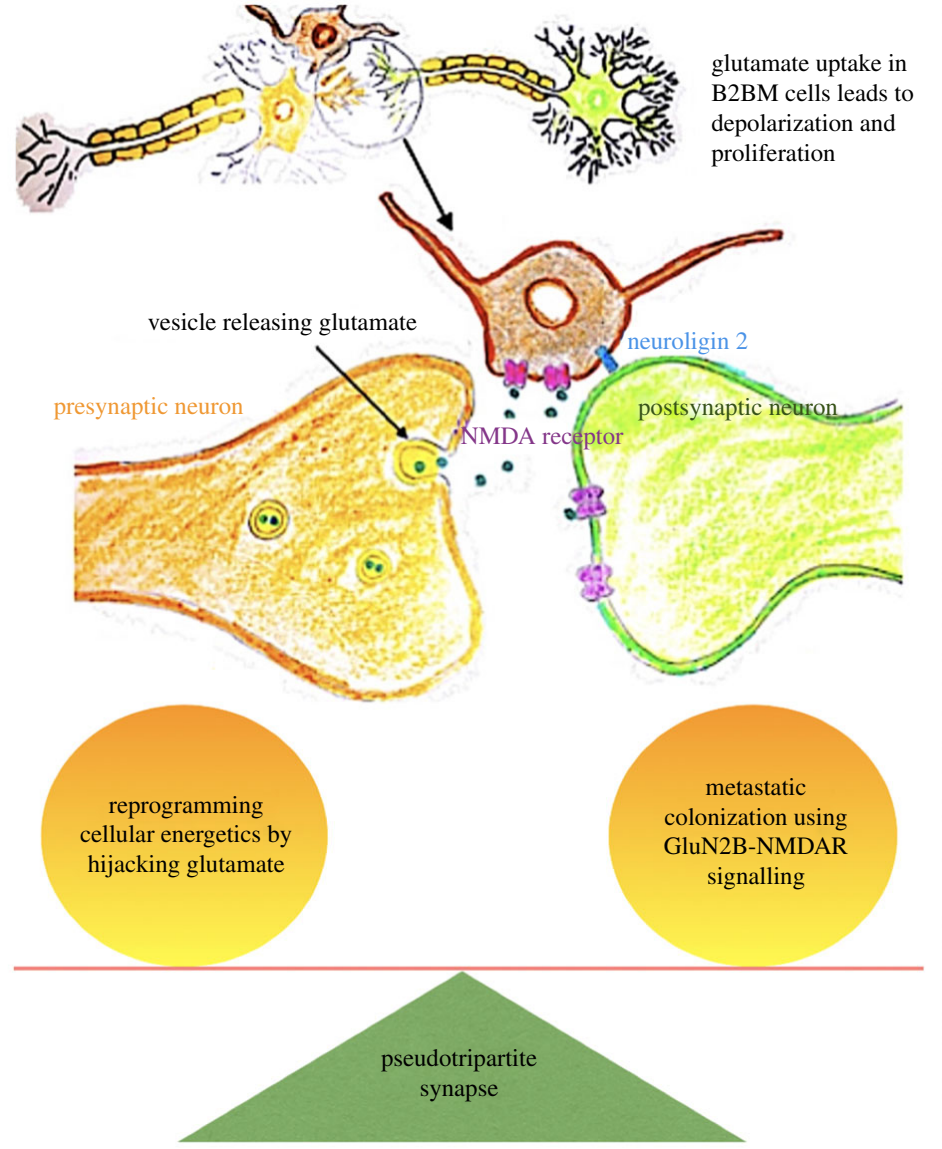
Parasitic tripartite synapse: B2BM colonization of brain using glutamate from the fake tripartite synapse, adapted based on [184].

Microscopic analysis of mouse B2BM models revealed a pseudo-tripartite synapse phenomenon. Finger-like projections emanated from the B2BM cells towards excitatory synapses, forming a fake tripartite synapse [184]. In normal neurophysiology, glutamate released from presynaptic neurons is endocytosed by postsynaptic neurons that express the glutamate receptor NMDAR, as well as by astrocytes which are located adjacent to the synaptic cleft [189]. This tripartite phenomenon is mimicked by the B2BM cells, which take the position of the astrocyte next to the synaptic cleft and use the glutamate from the presynaptic neuron to promote further metastasis and colonization in the brain (figure 13).

Modulation of GluN2B expression demonstrated that NMDAR signalling was not necessary for the initial seeding of breast tumour cells to the brain but, rather, was critical for the proliferation of B2BM cells [184]. Thus, B2BM cells effectively tune the neural niche to their advantage without disrupting the existing synaptic infrastructure. Tumour cell–astrocyte gap junctions can also be co-opted to promote brain metastasis via 2′3′-cyclic GMP-AMP (cGAMP)-mediated signalling. This can potentially be disrupted by the gap junction modulator meclofenamate, which has oral bioavailability and can pass through the blood–brain barrier [190]. Similarly, based on the inference that B2BM cells co-opt NMDAR signalling for metastatic progression in the brain [184], one could exploit the parasitic tripartite synapse for therapeutic and diagnostics purposes. One approach might be to repurpose memantine, an NMDAR antagonist used for treating Alzheimer's disease, to curtail the progression of B2BM in TNBC patients. Furthermore, radiolabelled glutamine can potentially be used for imaging triple-negative breast cancer brain metastasis [191].

### 6.6. Nerves and the tumour microenvironment

Nerves can also play a role in immune evasion during tumourigenesis by orchestrating an immune-suppressive tumour microenvironment. β2-adrenergic receptor signalling by adrenergic nerves can inhibit lymphocyte egress, effectively reducing the recruitment of antigen primed T cells [192]. Manipulation of autonomic nerves using a novel viral-vector based neuro-engineering technique revealed accelerated breast cancer progression with sympathetic nerve stimulation in tumours, while local sympathetic denervation curtailed tumour growth and reduced the expression of immune checkpoint molecules, such as programmed death-1 (PD-1) and PD-L1, as well as FOXP3, that mediates immunosuppression [193]. Such a strategy of the localized intervention targeting neural input, using genetic neuro-engineering techniques, may hold promise to stimulate the immune system while offsetting the deleterious side-effects of the systemic use of checkpoint inhibitors.

Nerves and neuronal signalling are an indispensable part of tumourigenesis, playing an active role in modulating the tumour microenvironment. They are involved in the recruitment of blood vessels to the tumour, control constriction/relaxation of blood vessels, alter the expression of immune checkpoint molecules and provide cues for proliferation to tumour cells, yet the nervous system has been largely disregarded in cancer therapeutics. Nerves and neuronal signalling are an enabling hallmark of cancer that provides tumours with a means of interacting with its microenvironment to facilitate metastatic progression. Future treatment regimens must work around the neural circuit to offer better control over tumour progression.

## 7. Conclusion

The understanding of cancer from a curse, to that of a heterogeneous group of diseases that lack the fundamental ability to respond to principal signals regulating proliferation, differentiation, and cell death is a phenomenal leap of understanding. From multiple resections without anaesthesia in ancient times, to targeted cancer therapeutics is certainly a remarkable feat of achievement. The Hallmarks of Cancer [194] marked the Millenium era for cancer researchers, laying the framework for honing our understanding of cancer as a disease. We present four novel hallmarks, the traits of which are the language which cancer cells use to interact with the microenvironment to facilitate proliferation and survival. We consider two additional core hallmarks: dedifferentiation/transdifferentiation and epigenetic dysregulation, alongside two enabling hallmarks: altered microbiome and altered neuronal signalling.

Seminal studies, we have discussed, overturned the unidirectional landscape of differentiation [9,10], yet the hallmark of dedifferentiation has long been ignored in the field of cancer therapeutics. The lineage plasticity conferred by the proposed hallmark of dedifferentiation, hijacked by tumour cells, can also be used for targeting tumour cells at their most vulnerable state to potentially transdifferentiate them to lineages which lack metastatic potential.

Two of the hallmarks proposed to confer a vantage point for therapeutic manipulation due to their reversible nature: epigenetic dysregulation and the microbiome. Epigenetic dysregulation provides numerous opportunities to intervene in cancer progression and development. For example, dietary factors can influence serum methionine levels, which in turn can affect histone methylation [195]. Microbiome dysbiosis can be manipulated by enhancing our ability to identify anti-carcinogenic (friend) and pro-carcinogenic (foe) among the microbiome. Microbiome composition must be integrated and used as a tool to enhance the outcome of therapeutics.

Finally, the hallmark of altered neuronal signalling consists of multiple clues to halt metastasis. The two factors which cancer cells use to design their microenvironment to their advantage are microbiome and nerves. Tumour cells use nerves to establish blood vessels and garner proliferative cues. Cancer can be associated with excruciating pain, a key being that cancer cells recruit numerous nerves, a trait that can be intercepted for pain management. Two modalities for managing the hallmark of altered neuronal signalling are either to include resection of nerves in surgical protocols for tumour management (significantly more challenging than resecting lymph nodes), or to target the nerve growth factor/localized intervention of neuronal signalling within the tumour microenvironment. Future studies may look into possibilities of targeting artemin which has an established role in the migration of sympathetic precursors [196,197].

Considering cancer as the conductor of a malign symphony and the hallmarks as the musicians, we need to tune our hearing to appreciate every key nuance of the piece. By identifying new performers, we can adapt our interventions, re-educating the orchestra and re-establishing the rhythm of life.

## Data accessibility

This article has no additional data.
